# Neural substrates of smoking and reward cue reactivity in smokers: a meta-analysis of fMRI studies

**DOI:** 10.1038/s41398-020-0775-0

**Published:** 2020-03-17

**Authors:** Xiao Lin, Jiahui Deng, Le Shi, Qiandong Wang, Peng Li, Hui Li, Jiajia Liu, Jianyu Que, Suhua Chang, Yanping Bao, Jie Shi, Daniel R. Weinberger, Ping Wu, Lin Lu

**Affiliations:** 1 Peking-Tsinghua Center for Life Sciences and PKU-IDG/McGovern Institute for Brain Research, Peking University, Beijing, 100871 China; 2 Peking University Sixth Hospital, Peking University Institute of Mental Health, Key Laboratory of Mental Health, Ministry of Health (Peking University), National Clinical Research Center for Mental Disorders (Peking University Sixth Hospital), Beijing, 100191 China; 3 National Institute on Drug Dependence and Beijing Key Laboratory of Drug Dependence, Peking University, Beijing, 100191 China; 4 Lieber Institute for Brain Development and Departments of Psychiatry, Neurology, and Neuroscience and McKusick Nathans Institute of Genetic Medicine, Johns Hopkins University School of Medicine, Baltimore, MD 21205 USA

**Keywords:** Neuroscience, Psychiatric disorders

## Abstract

Smoking is partly attributed to alterations of reward processing. However, findings on the neurobiological mechanisms that underlie smoking-related and smoking-unrelated reward processing in smokers have been inconsistent. Neuroimaging experiments that used functional magnetic resonance imaging (fMRI) and reported brain responses to smoking-related cues and nonsmoking reward-related cues in smokers and healthy controls as coordinates in a standard anatomic reference space were identified by searching the PubMed, Embase, and Web of Science databases up to December 2018. Three meta-analyses were performed using random-effect nonparametric statistics with Seed-based d Mapping software, with brain activity contrast from individual studies as the input. The striatum showed higher activation in response to smoking-related cues compared with neutral cues in 816 smokers from 28 studies and lower activation in response to nonsmoking reward-related cues in 275 smokers compared with 271 healthy control individuals from 13 studies. The relative reactivity of the putamen to smoking-related cues increased in 108 smokers compared with 107 healthy controls from seven studies. Meta-regression showed that smokers with a greater severity of nicotine dependence exhibited less engagement of the striatum in response to both smoking-related cues and nonsmoking reward-related cues. The present results reveal the disruption of reward system function in smokers and provide new insights into diverging theories of addiction. With the escalation of nicotine dependence, nicotine appears to exert dynamic effects on reward processing, based on incentive sensitization theory and reward deficiency syndrome theory.

## Introduction

Cigarette smoking is a chronic relapsing disorder and one of the leading causes of preventable disease and death. More than 16 million Americans live with smoking-related diseases, and >480,000 people die from smoking every year. Approximately 1 in 5 deaths are attributable to smoking^1^. Given the current situation, yearly smoking-related deaths are expected to rise to 8 million by 2030^2^. Although most smokers report a desire to quit, the majority of attempts fail within 10 days^3^. Numerous studies reported that craving that is induced by smoking-related cues is responsible for the failure to quit and high rates of relapse^4,5^. One core characteristic of addiction is the disruption of reward processing^6^, which influences decision-making and leads to smoking behavior^7,8^. The relationship between alterations of reward processing and nicotine dependence has been well demonstrated, but no consensus has been reached on the neurobiological substrates that underling smoking-related and nonsmoking reward-related processing.

According to incentive sensitization theory, repeated pairings between smoking-related cues (e.g., cigarette packs, lighters, and smoking-related pictures and videos) and smoking itself increase the dopaminergic response to these nonpharmacological cues, ultimately conferring heightened incentive properties to these stimuli^9,10^. Neural systems that subserve reward-related learning, primarily represented in the striatum, are hijacked by reward cues and related contexts that predict the availability of smoking, resulting in higher responsivity to smoking-related cues and relatively lower responsivity to nonsmoking reward-related cues. Using functional magnetic resonance imaging (fMRI), based on the contrast of blood oxygen level-dependent (BOLD) signals, several studies investigated brain activation in response to smoking-related stimuli compared with neutral cues in smokers. Using a cue exposure task, Baker et al. found that smokers exhibited heightened reward responsiveness to cigarette rewards relative to monetary rewards^11^. A cohort study that used an incentive-processing task found that individuals who smoked more cigarettes per day exhibited lower right nucleus accumbens reactivity to a nonsmoking reward^12^.

In contrast to incentive sensitization theory, reward deficiency syndrome theory proposes that addicts exhibit general deficits in the recruitment of brain reward pathways, resulting in the chronic hypoactivation of these circuits in response to both drug- and nondrug-related reward^13^. Several studies support this proposition. For example, dependent smokers exhibited equivalently low reward system activity in response to both reward types^14^. An fMRI study revealed that smokers exhibited lower brain reactivity, especially in reward-related brain regions (e.g., caudate and putamen), to nontobacco reward-related stimuli (e.g., favorite food) compared with nonsmokers^15^. Another study investigated the association between the severity of nicotine dependence and the response to monetary rewards and found that the intensity of nicotine craving among smokers was linked to lower sensitivity to nondrug-related rewards^16^. The threshold of reward has been shown to increase as addiction develops, expressed by higher drug tolerance.

These divergent results may be attributable to different cue exposure paradigms and different fMRI parameters. Furthermore, small sample sizes hamper the ability to distinguish general activation patterns. A qualitative meta-analysis by Engelmann et al. (2012) reported activation of the precuneus, cingulate gyrus, dorsal and medial prefrontal cortex, insula, and dorsal striatum when smokers were presented with smoking-related cues compared with neutral cues^17^. However, these authors only included smokers and did not make comparisons with healthy controls. A more complete analysis is needed to understand the neural processing of smoking-related cues in smokers and differences from healthy controls. The present study investigated specific smoking-related activation patterns compared with neutral cues in smokers and compared brain activity in response to smoking-related cues between smokers and healthy controls. We also compared brain activation in response to nonsmoking reward-related cues in smokers and healthy controls. We sought to provide an overall reward processing profile of neural substrates in smokers.

## Methods and materials

### Study selection

**Fig. 1:**
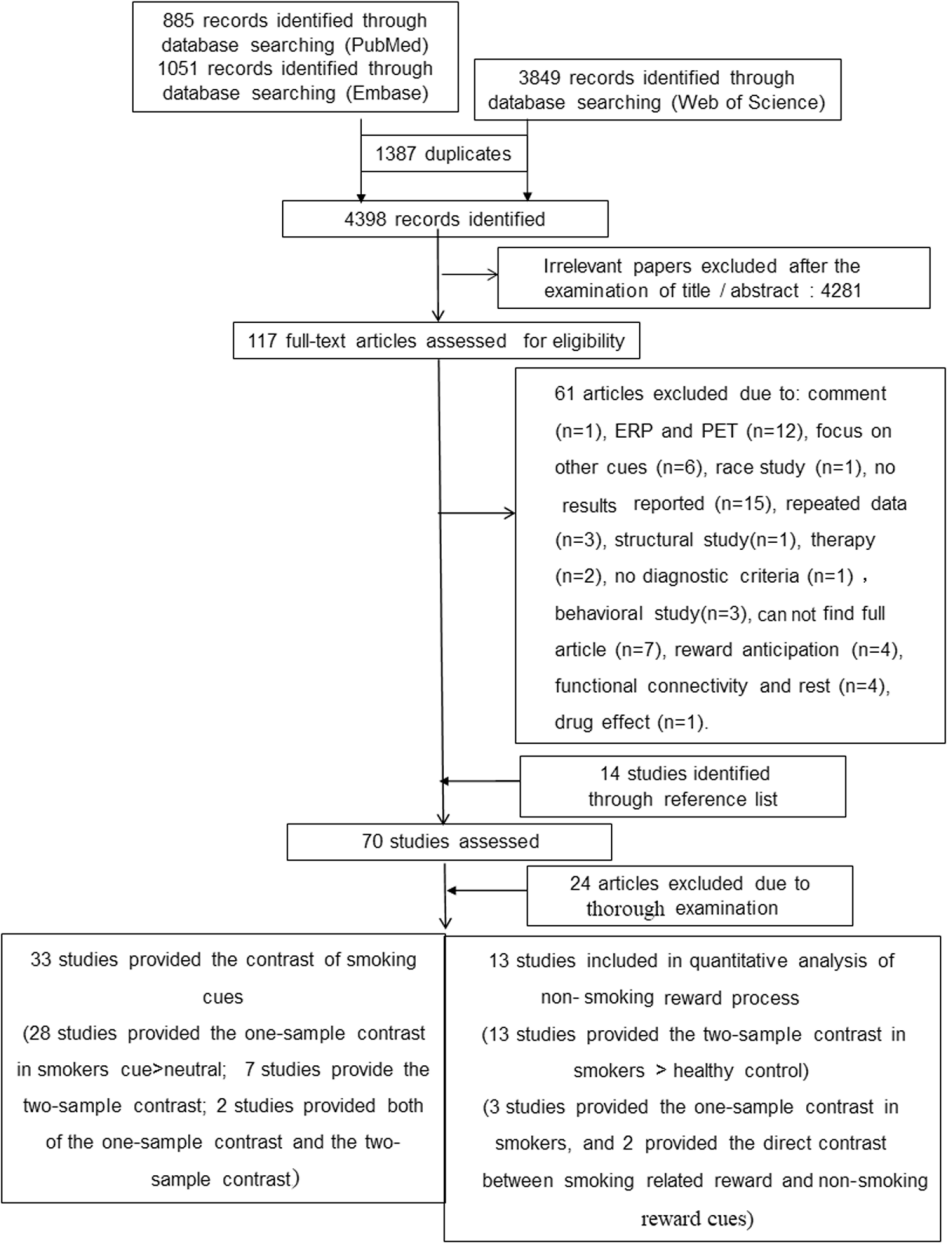
Flow chart of the identification of eligible studies.

Candidate studies for the cue reactivity meta-analysis were identified by searching the PubMed (Medline), Embase, and Web of Science databases. The combination of search terms included (i) smok* (smoking, smoker, cigarette, tobacco, nicotine) AND (ii) fMRI (e.g., neuroimaging, brain imaging, BOLD, fMRI, MRI, brain reactivity, neutral reactivity) AND (cue reactivity [cue, smoking-related, smoking cues] OR reward [money, reward, monetary, gain]). The time span for the included studies was from 1 January 2000 to 31 November 2018. The reference lists of the publications and related reviews^17–19^ were also examined to identify additional studies. A study was eligible if it (i) was a task-related fMRI study, (ii) compared smoking-related cues with neutral cues (all modalities pooled) or involved nonsmoking reward-related cues, (iii) provided peak coordinates in Montreal Neurological Institute (MNI) space or Talairach space, and (iv) measured nicotine dependence severity using the Fagerström Test for Nicotine Dependence (FTND). The studies that were included in the present meta-analysis did not necessarily include two groups (only smokers or smokers and healthy controls). The searches identified 33 articles that involved fMRI and smoking cues (two studies reported brain reactivity to smoking-related cues in smokers and comparisons between smokers and healthy controls; 26 studies only reported brain reactivity to smoking-related cues in smokers; five studies only reported comparisons between smokers and healthy controls), resulting in 28 publications that were included in the meta-analysis that examined brain reactivity to smoking-related cues in smokers and seven studies that examined brain reactivity to smoking-related cues in smokers compared with healthy controls. We also identified 13 publications for nonsmoking reward processing in smokers compared with healthy controls. The detailed process of identifying eligible studies and the reasons for exclusion are presented in Fig. 1.

### Data analysis

All coordinate-based meta-analyses were performed using anisotropic effect-size Seed-based d Mapping (SDM, version 5.1.4, http://www.sdmproject.com, formerly “Signed Differential Mapping”)^20,21^. A noteworthy feature of SDM is that it considers studies with positive, negative, and nonsignificant results simultaneously, leading to more precise estimates. It can also provide information about between-study heterogeneity. The software first recreates the study maps of the effect size of differences in BOLD responses between patients and control subjects by converting the *t* value of each peak to Hedges’ effect size and then applies an anisotropic nonnormalized Gaussian kernel so that voxels that are more correlated with the peak have higher effect sizes. Maps are combined with a standard random-effects model, taking into account sample size, intrastudy variability, and between-study heterogeneity. In the SDM Z map, studies with lower variability and a larger sample size made a stronger contribution. Statistical inference was empirically estimated using permutation statistics (i.e., randomization of effect sizes across voxels). Previous studies recommended that 20 permutations lead to stable estimates. In the present study, all of the analyses that are reported were based on 50 permutations^22^. The statistical heterogeneity of individual clusters was examined using a random-effects model. All thresholds were *p* < 0.005, uncorrected with peak height *z* ≥ 1 and cluster extent = 10 voxels. For each significant patient-control comparison, Egger’s test was used to assess the asymmetry of the funnel plot as a measure of potential publication bias^21,23^. Three different meta-analyses were conducted. First, smoking cue-induced brain activity was compared with the response to neutral cues in smokers. To better understand the influence of nicotine state (abstinent or satiated) on brain reactivity to smoking-related cues, two separate subgroup analyses for abstinent state and satiated state were conducted, and similarities or differences between states were explicitly tested. Second, brain activation in response to smoking-related cues in smokers was then compared with healthy controls. Third, brain activation in response to nonsmoking reward-related cues in smokers was compared with healthy controls. The relationship between the severity of nicotine dependence (FTND scores) and reactivity of reward-related brain regions to smoking-related and nonsmoking reward-related cues was examined using meta-regression.

## Results

### Literature search and characteristics of the included studies

As shown in Fig. 1, a total of 46 papers were retrieved. Twenty-eight of these studies met the inclusion criteria, providing a sample size of 816 smokers (317 female) to investigate brain activity in response to smoking-related cues in smokers. Seven studies reported a comparison between smokers and healthy controls, providing a sample size of 108 smokers (32 female) and 107 healthy controls (37 female). The nonsmoking reward cue processing meta-analysis included 13 studies that evaluated the neural process of reward-related cue exposure, with a sample size of 275 smokers (114 female) and 271 healthy controls (102 female). The specific details of these studies, including demographic information, are shown in Supplementary Table S1.

### Main meta-analyses of cue-reactivity in smokers

**Fig. 2:**
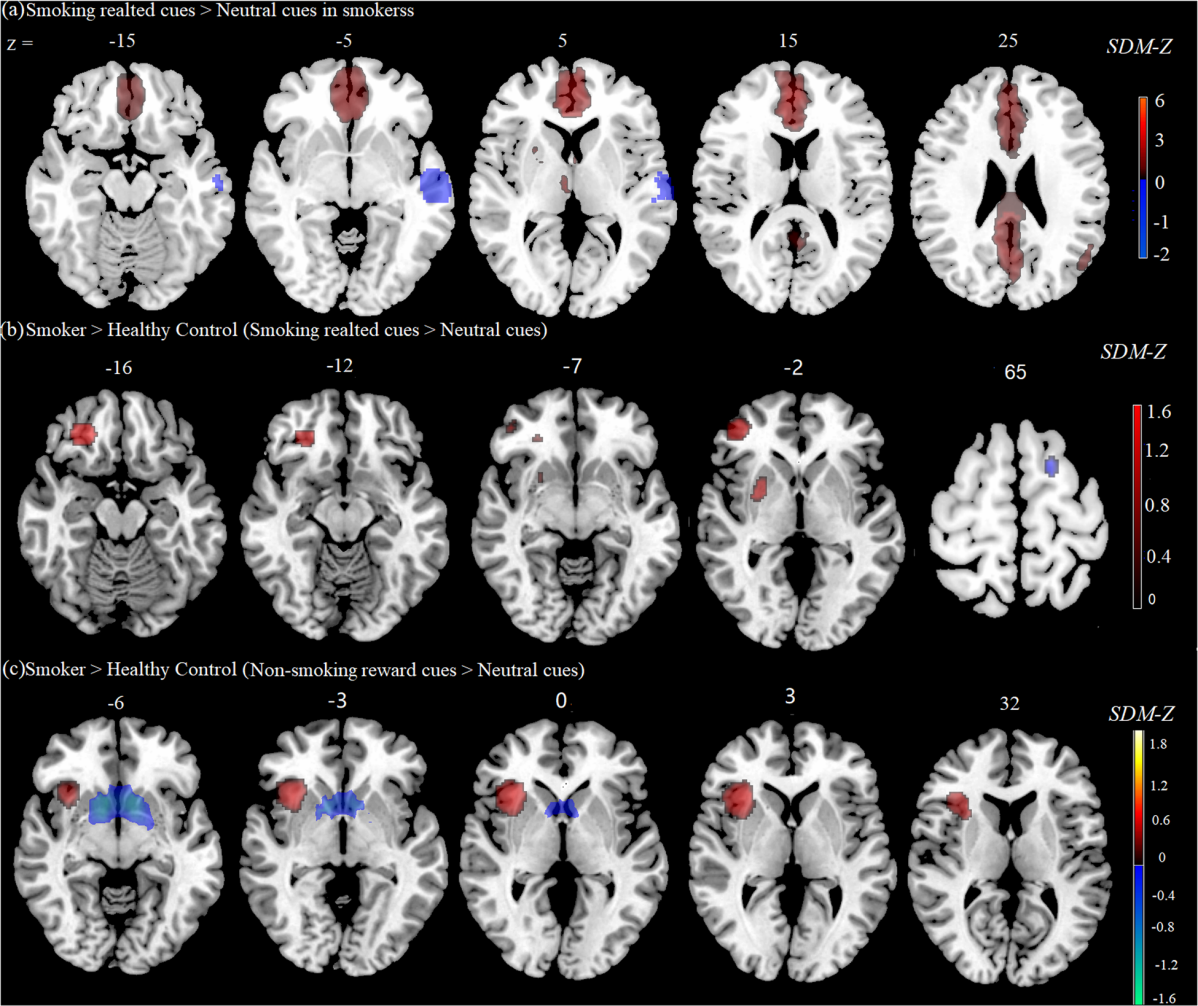
Brain regions showed significant differences in response to smoking-related cues or nonsmoking reward-related cues based on the meta-analyses. **a** Brain regions that showed significant differences between smoking-related cues and neutral cues in smokers. **b** Brain regions that showed significant differences between smokers and healthy controls in response to smoking-related cues. **c** Brain regions that showed significant differences between smokers and healthy controls in response to nonsmoking reward-related cues.

Our first meta-analysis included the activation coordinates from all 28 studies. This analysis revealed five statistically significant clusters of smoking *minus* neutral activation in smokers (Table 1, Fig. 2a). The largest cluster was located within the left cingulate gyrus, including the anterior and posterior areas, and most of the significantly active voxels were located within the anterior cingulate gyrus. Other areas with a large concentration of clusters were the left angular gyrus and right thalamus. Smaller but theoretically important clusters of activation were detected in and around the right striatum. The increase in activation of the bilateral cingulate gyrus in response to smoking-related cues was consistently reported in smokers. To better address the influence of nicotine state (abstinent or satiated) on brain reactivity to smoking-related cues, a subgroup meta-analysis was also conducted. The two separate analyses of all abstinent-state and satiated-state studies also revealed a consistent increase in activation of the anterior cingulate gyrus (ACC) and right striatum in response to smoking-related cues in smokers compared with neutral cues (for details, see Supplementary Table S2 and Table S3).

**Table 1:** Suprathreshold clusters from activation likelihood estimation meta-analysis.

Brain region	MNI coordinates	SDM-Z	*p*	Voxels
*Smoking cues minus neutral cues in smokers*				
Left anterior cingulate/paracingulate gyri, BA 32	−2, 50, 12	5.659	~0	8535
Left angular gyrus, BA 39	−52, −64, 24	3.213	0.00091	177
Right thalamus	4, −16, 4	3.364	0.00045	71
Corpus callosum	−2, 2, 6	3.058	0.00181	34
Right striatum	20, 10, 2	3.153	0.00119	27
Left middle temporal gyrus, BA 22	−54, −16, −2	−1.014	0.00024	621
*Smokers minus Healthy controls in response to smoking cues*				
Right middle frontal gyrus, BA 46	38, 48, 4	1.506	0.00016	350
Right middle frontal gyrus, orbital part, BA 11	26, 38, −18	1.502	0.000186	264
Right lenticular nucleus, putamen	28, 4, 0	1.235	0.002276	71
Left superior frontal gyrus, dorsolateral, BA 6	−20, 6, 66	−1.037	0.001216	34
*Smokers minus Healthy controls in response to nonsmoking reward cues*				
Right inferior parietal (excluding supramarginal and angular) gyri, BA 40	36, −54, 52	1.378	0.000006	826
Right insula, BA 47	34, 22, 0	1.377	0.000006	755
Right inferior frontal gyrus, opercular part, BA 44	52, 18,36	1.065	0.000275	296
Left striatum	−8, 12, −10	−2.662	~ 0	2987

Coordinates are given for the maximally significant voxel in each area, where *x* defines the lateral placement from the midline (left = negative), *y* defines the anteroposterior displacement relative to the anterior commissure (posterior = negative) and *z* defines the vertical position relative to the anteroposterior commissural line (down = negative). The coordinates are in Montreal Neurological Institute (MNI) space.

### Main meta-analysis of comparison of smoking cue-induced brain reactivity between smokers and healthy controls

After combining the activation coordinates from seven studies, four statistically significant clusters of smokers *minus* healthy controls were found (Table 1, Fig. 2b). Many of these clusters that showed higher activity in smokers were located within the right middle frontal gyrus. Other areas with a large concentration of clusters included the orbital frontal gyrus and putamen. The lower activity in smokers (compared with healthy controls) when processing smoking-related cues was located within the right dorsolateral prefrontal cortex.

### Main meta-analysis of comparison of activity in response to nonsmoking reward-related cues between smokers and healthy controls

The meta-analysis of nonsmoking reward-related activity included activation coordinates from all 13 studies. Four statistically significant clusters were found (Table 1, Fig. 2c), three of which showed higher activation in smokers compared with healthy controls and one of which showed lower activity. Most of the clusters that showed higher activation were located within the right inferior parietal gyrus and frontal gyrus. Other areas with a large concentration of clusters were located within the insula and inferior prefrontal cortex. Importantly, one large cluster that showed lower activation was located within reward circuitry, specifically the right striatum.

### Meta-regression results

**Fig. 3:**
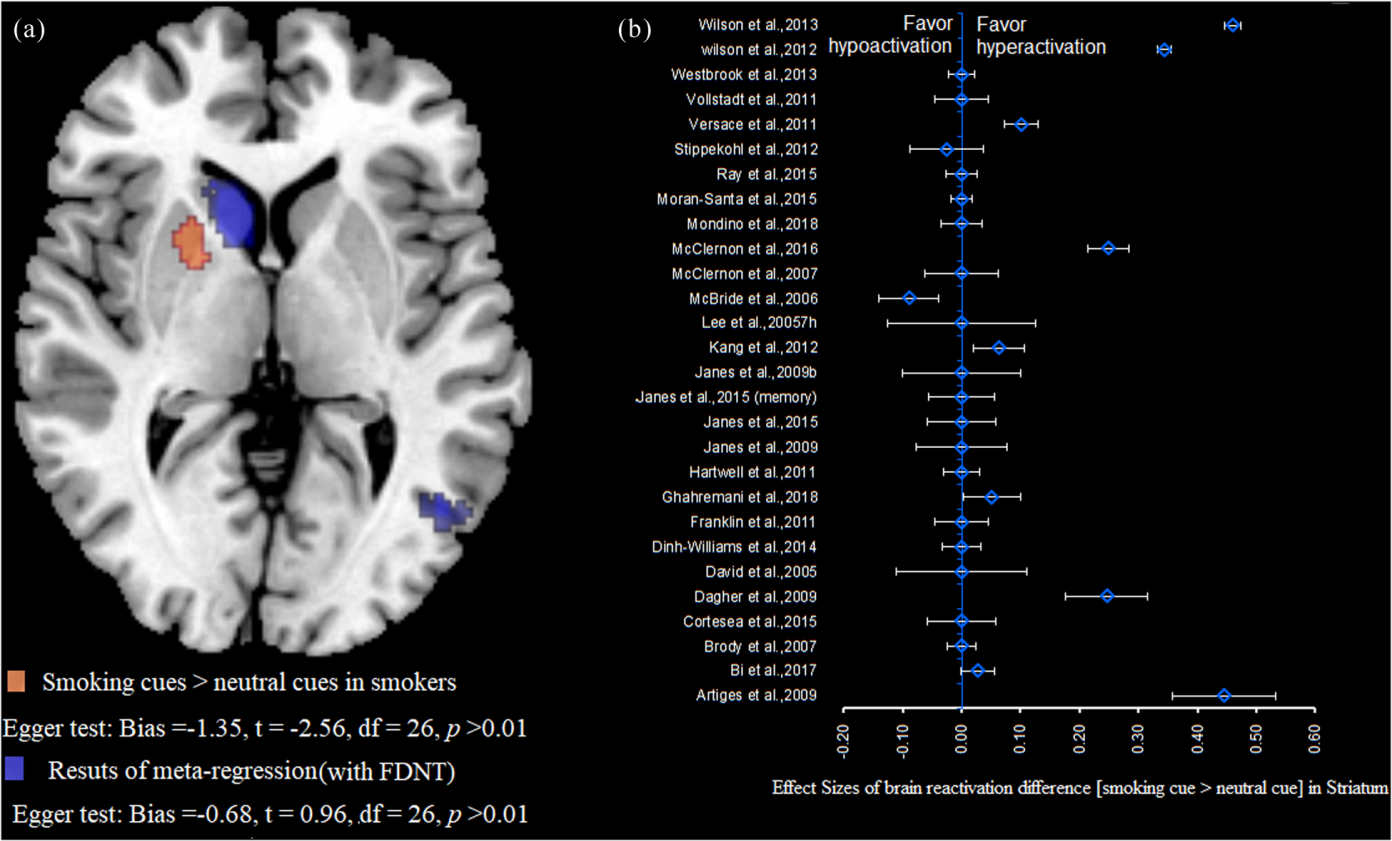
Results of meta-regression in brain reactivity to smoking-related cues in smokers. **a** The cluster within the striatum that showed greater activation in response to smoking-related cues compared with neutral cues and the cluster within the striatum that showed differences in the meta-regression analysis with FTND score as a factor. **b** Forest plot of the mean ± variance of effect sizes for activation differences in the striatum, estimated from individual studies with smoking-related cue exposure.

**Fig. 4:**
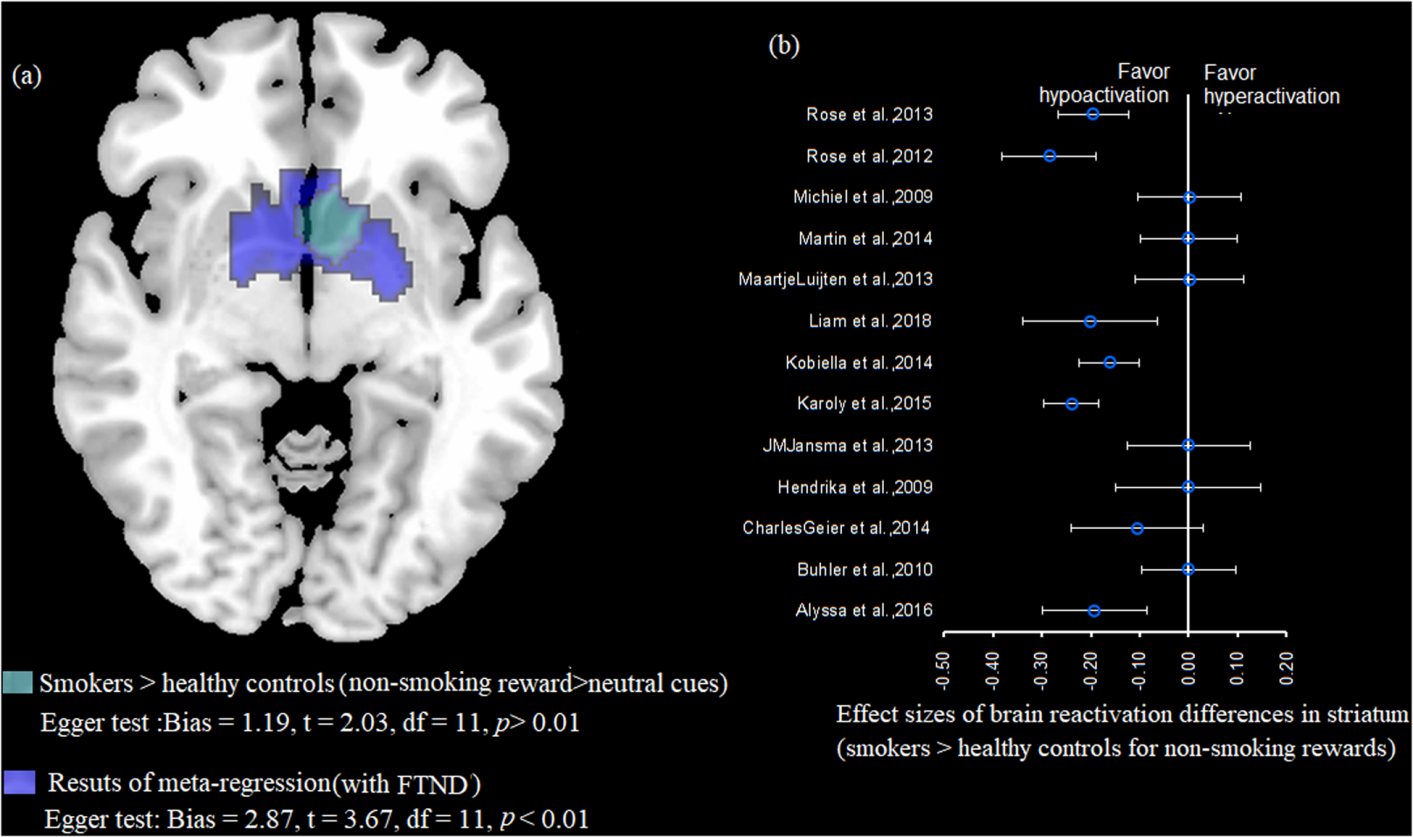
Results of meta-regression in brain reactivity to nonsmoking reward-related cues in smokers. **a** The cluster within the striatum that showed greater activation in response to nonsmoking reward-related cues in smokers compared with healthy controls and the cluster within the striatum that showed differences in the meta-regression analysis with FTND score as a factor. **b** Forest plot of the mean ± variance of effect sizes for group differences in the striatum, estimated from individual studies with nonsmoking reward-related cue exposure.

To examine the effects of the severity of nicotine dependence on reward processing in smokers, meta-regression analyses were performed. The results revealed a significant relationship between FTND scores and activation of the striatum. This indicates that with greater dependence, activation of the striatum in response to reward cues decreased (i.e., both smoking-related cues (Fig. 3, Supplementary Table S4) and nonsmoking reward-related cues (Fig. 4, Supplementary Table S5)). Interestingly, we found a significant positive correlation between the severity of nicotine dependence and reactivation of the anterior cingulate gyrus in response to nonsmoking reward-related cues, suggesting that greater involvement of the executive control brain regions in response to reward cues is associated with the greater severity of nicotine dependence.

### Sensitivity analyses and publication bias tests

Jackknife sensitivity analyses were conducted to evaluate the robustness of the results. In our analysis, alterations were replicable in all combinations of studies. Leave-one-out validation indicated that regardless of which study was left out, the meta-analysis consistently identified greater activation of the anterior cingulate gyrus in smokers in response to smoking-related cues. All of the combinations consistently reported lower activation of the striatum in response to nonsmoking reward-related cues in smokers compared with healthy controls. The results of the Jackknife sensitivity analyses for the striatum are presented in the Supplementary Materials (Supplementary Tables S6–8).

Publication bias is generally the tendency of researchers, journal editors, and corporate entities to manage the reporting of experimental findings that are “significant” differently from findings that are “nonsignificant.” This then leads to bias in the overall published literature toward only “statistically significant” effects. Publication bias in the present meta-analyses was tested using Egger’s regression approach. For visual inspection, funnel plots were also generated (Supplementary Figure S1).

## Discussion

We conducted three meta-analyses of fMRI studies of smoking-related cue exposure and nonsmoking reward-related cue exposure in smokers. Activation of the striatum and anterior cingulate gyrus increased in response to smoking-related cues vs. neutral cues in smokers. The putamen showed greater activation in smokers vs. healthy controls when processing smoking-related cues. The striatum showed lower activation in smokers vs. healthy controls in response to nonsmoking reward-related cues. The meta-regression analysis showed that sensitivity of the reward system was disrupted as the severity of nicotine dependence increased, with lower activation of the striatum in response to smoking-related cues and nonsmoking reward-related cues in smokers with higher FTND scores.

In response to smoking-related cues compared with neutral cues in smokers, the cingulate gyrus was the most consistently activated. The cingulate gyrus is a critical region of the limbic system. Previous studies have shown that the anterior cingulate gyrus receives projections from structures that process rewards, including the orbitofrontal cortex, striatum, and mesolimbic dopamine system^24^. Lesions of the anterior cingulate gyrus impaired reward learning and behavioral adjustments that were related to changes in reward^25^. The present study found a robust positive correlation between reactivation of the anterior cingulate gyrus in response to nonsmoking reward-related cues and the severity of nicotine dependence. The posterior cingulate gyrus is a critical center of the default mode and attentional networks^26^. Neuroimaging studies have shown that it is activated during visual tasks when a monetary incentive is involved, essentially functioning as a neural interface between motivation-related areas and the top-down control of visual attention^27,28^. Increases in activation of the prefrontal cortex are related to executive control, suggesting that smokers need to exert extra control of smoking urges when exposed to smoking-related cues. The increase in activation of the cingulate gyrus and prefrontal cortex in response to smoking-related cues in smokers suggests an increase in the distribution of attention to smoking-related cues in smokers. This is consistent with previous studies that found greater attentional bias toward drug-related cues in smokers. For example, smokers maintained their gaze on smoking-related cues for a longer period of time compared with control cues^29,30^. This result might also be partially explained by incentive sensitization theory, which proposes that repeated associations between drug-related cues and addictive drugs increase the dopaminergic response to these essentially neutral cues, ultimately causing pathological incentive motivation (“wanting”) for these stimuli^9,10^.

Compared with healthy controls, the activation of reward circuitry significantly increased in response to smoking-related cues and significantly decreased in response to nonsmoking reward-related cues in smokers, thus supporting incentive sensitization theory. After repeated associations with smoking, the nonpharmacological neutral stimulus acquires the properties of an incentive reward. Greater activation in the caudate nucleus and cingulate gyrus is consistently found, with the notion that the presence of smoking-related cues increases nicotine/tobacco seeking and hence relapse rates^31^. The striatum and medial prefrontal cortex have been shown to be activated by smoking-related cues, demonstrating the ability of smoking-related stimuli to engage reward-related circuitry. Our meta-analysis showed relatively consistent activation of the prefrontal cortex and striatum, further supporting incentive sensitization theory.

For nonsmoking reward processing, the significant decrease in activation of the caudate in smokers compared with healthy controls is consistent with reward deficiency syndrome theory. This theory of motivation proposes that addicts have a general deficit in the recruitment of brain reward pathways, resulting in chronic hypoactivation of these circuits in response to both drug- and nondrug-related reward^13^. Previous studies have consistently reported greater activation of the striatum in response to monetary rewards. The striatum has been shown to be involved in reward prediction and the subjective value of rewards^32^. In substance-dependent individuals, the incentive value of nondrug reward is lost, leading to lower activation in the striatum during the processing of monetary and other natural rewards. The decrease in activation of the striatum in response to nondrug rewards has also been observed in opioid addicts^33^, cocaine addicts^34^, and alcohol abusers^35^. However, the finding of lower activation of the striatum in response to nondrug rewards contradicts the recent meta-analysis by Luijten et al.^22^. This discrepancy might be associated with three important differences between these two studies. First, the present study focused on nonsmoking reward-related cues regardless of the type of cue. We pooled monetary and juice cues, whereas the study by Luijten focused only on monetary cues. Second, the present study focused on reward-related cues and reward outcome phases and did not include an anticipatory phase; therefore, the nonsmoking reward-related cues represented the presence of reward outcomes. Third, the present study focused only on nicotine addiction and did not include other addictions. A previous study performed a direct comparison between food cues and smoking cues and found greater activation of the orbitofrontal cortex and cingulate gyrus in response to smoking cues compared with food cues^36^. The greater engagement of attentional networks and reward-related brain regions during exposure to smoking-related cues is consistent with the present results.

Interestingly, the meta-regression revealed lower activation of the striatum in response to both types of rewards in smokers who had higher FTND scores. This seems to contradict incentive sensitization theory, thus supporting reward deficiency syndrome theory instead. Combining the main results of the meta-analyses and meta-regression, incentive sensitization theory and reward deficiency syndrome theory do not appear to conflict with each other when considering the severity of dependence. Nicotine first appears to hijack the reward system and gain incentive value, which is consistent with incentive sensitization theory. As the severity of nicotine dependence increases, sensitivity of the reward system is disrupted, which could be explained by reward deficiency syndrome theory. These two theories could be combined to interpret dissociable activation of the striatum that is observed in smokers. The disruption of reward reactivity to reward-related cues coincides with the development of drug tolerance, meaning that the same dose of nicotine fails to induce the same activation as nicotine dependence develops. Further studies are needed to verify dynamic alterations of the reward system that occur in other kinds of drug dependence and investigate the relationship between reward processing deficits and the severity of drug dependence.

The present study has several limitations that are inherent to all meta-analyses. First, meta-analyses that are based on peak and effect sizes use data from published studies rather than raw statistical brain maps, thus decreasing accuracy of the results. Second, the results of the present study should be interpreted with caution when considering inter-study heterogeneity. Different studies adopted different statistical thresholds and multiple-comparison corrections.

In conclusion, the reward system appears to be hijacked by the initiation of nicotine dependence, expressed as greater activation of the reward system in response to smoking-related cues and lower activation of the reward system in response to nonsmoking reward-related cues. However, activation in response to both types of cues decreased as the severity of nicotine dependence increased, indicating general disruption of sensitivity of the reward system. The present study further reveals the dynamic nature of reward system alterations in smokers and provides new insights into disentangling the divergence between incentive sensitization theory and reward deficiency syndrome theory to explain addiction.

## Supplementary information

Supplementary Material

## References

[1] Jamal A (2018). Current cigarette smoking among adults in the United States, 2016. Morb. Mortal. Wkly. Rep..

[2] World Health Organization. (2008). WHO Report on the Global Tobacco Epidemic, 2008. The MPOWER package..

[3] Piasecki TM (2006). Relapse to smoking. Clin. Psychol. Rev..

[4] Janes AC (2010). Brain reactivity to smoking cues prior to smoking cessation predicts ability to maintain tobacco abstinence. Biol. Psychiatry.

[5] Field M, Marhe R, Franken IH (2014). The clinical relevance of attentional bias in substance use disorders. CNS Spectr..

[6] Hyman SE, Malenka RC, Nestler EJ (2006). Neural mechanisms of addiction: the role of reward-related learning and memory. Annu. Rev. Neurosci..

[7] Schneider S (2012). Risk taking and the adolescent reward system: a potential common link to substance abuse. Am. J. Psychiatry.

[8] Volkow ND (2010). Addiction: decreased reward sensitivity and increased expectation sensitivity conspire to overwhelm the brain’s control circuit. BioEssays.

[9] Robinson TB, Kent C (1993). The neural basis of drug craving: an incentive-sensitization theory of addiction. Brain Res. Brain Res. Rev..

[10] Robinson TE, Berridge KC (2008). The incentive sensitization theory of addiction: some current issues. Philos. Trans. R. Soc. Lond. B. Biol. Sci..

[11] Baker TE (2017). Reversing the atypical valuation of drug and nondrug rewards in smokers using multimodal neuroimaging. Biol. Psychiatry.

[12] Peechatka AL, Janes AC (2017). Association between reward reactivity and drug use severity is substance dependent: preliminary evidence from the human connectome project. Nicotine Tob. Res..

[13] Blum K (2000). Reward deficiency syndrome: a biogenetic model for the diagnosis and treatment of impulsive, addictive, and compulsive behaviors. J. Psychoact. Drugs.

[14] Buhler M (2010). Nicotine dependence is characterized by disordered reward processing in a network driving motivation. Biol. Psychiatry.

[15] Jastreboff AM (2015). Blunted striatal responses to favorite-food cues in smokers. Drug Alcohol Depend..

[16] Peechatka AL, Whitton AE, Farmer SL, Pizzagalli DA, Janes AC (2015). Cigarette craving is associated with blunted reward processing in nicotine-dependent smokers. Drug Alcohol Depend..

[17] Engelmann JM (2012). Neural substrates of smoking cue reactivity: a meta-analysis of fMRI studies. Neuroimage.

[18] Versace F (2017). Beyond cue reactivity: non-drug-related motivationally relevant stimuli are necessary to understand reactivity to drug-related cues. Nicotine Tob. Res..

[19] Azizian A, Monterosso J, O’Neill J, London ED (2009). Magnetic resonance imaging studies of cigarette smoking. Handb. Exp. Pharmacol..

[20] Radua J, Mataix-Cols D (2009). Voxel-wise meta-analysis of grey matter changes in obsessive compulsive disorder. Br. J. Psychiatry.

[21] Radua J (2012). A new meta-analytic method for neuroimaging studies that combines reported peak coordinates and statistical parametric maps. Eur. Psychiatry.

[22] Luijten M, Schellekens AF, Kuhn S, Machielse MWJ, Sescousse G (2017). Disruption of reward processing in addiction: an image-based meta-analysis of functional magnetic resonance imaging studies. JAMA Psychiatry.

[23] Norman LJ (2016). Structural and functional brain abnormalities in attention-deficit/hyperactivity disorder and obsessive-compulsive disorder: a comparative meta-analysis. JAMA Psychiatry.

[24] Apps MA, Rushworth MF, Chang SW (2016). The anterior cingulate gyrus and social cognition: tracking the motivation of others. Neuron.

[25] Critchley HD (2010). Neural mechanisms of autonomic, affective, and cognitive integration. J. Comp. Neurol..

[26] Leech R, Braga R, Sharp DJ (2012). Echoes of the brain within the posterior cingulate cortex. J. Neurosci..

[27] Small DM, Gitelman DK, Bloise SM, Parrish T, Mesulam MM (2005). Monetary incentives enhance processing in brain regions mediating top-down control of attention. Cereb. Cortex.

[28] Engelmann JB, Damaraju E, Padmala S, Pessoa L (2009). Combined effects of attention and motivation on visual task performance: transient and sustained motivational effects. Front. Hum. Neurosci..

[29] Field M, Mogg K, Bradley BP (2004). Eye movements to smoking-related cues: effects of nicotine deprivation. Psychopharmacology.

[30] Mogg K, Bradley BP, Field M, De Houwer J (2003). Eye movements to smoking-related pictures in smokers: relationship between attentional biases and implicit and explicit measures of stimulus valence. Addiction.

[31] Conklin CA (2015). Examining the relationship between cue-induced craving and actual smoking. Exp. Clin. Psychopharmacol..

[32] Peters J, Büchel C (2010). Neural representations of subjective reward value. Behav. Brain. Res..

[33] Martin-Soelch C (2010). Changes in reward-induced brain activation in opiate addicts. Eur. J. Neurosci..

[34] Goldstein RZ (2007). Is decreased prefrontal cortical sensitivity to monetary reward associated with impaired motivation and self-control in cocaine addiction?. Am. J. Psychiatry.

[35] Beck A (2009). Ventral striatal activation during reward anticipation correlates with impulsivity in alcoholics. Biol. Psychiatry.

[36] Claus ED, Blaine SK, Filbey FM, Mayer AR, Hutchison KE (2013). Association between nicotine dependence severity, BOLD response to smoking cues, and functional connectivity. Neuropsychopharmacology.

