# Supplementary material for: Neural substrates of smoking and reward cue reactivity in smokers: a meta-analysis of fMRI studies
Source: Transl Psychiatry. 2020 Mar 17;10:97. doi: 10.1038/s41398-020-0775-0 (PMC7078287; doi:10.1038/s41398-020-0775-0)
Supplement: Supplementary file 1 — Supplementary Material [file 41398_2020_775_MOESM1_ESM.docx]

# February 15, 2020

# Supplementary Materials

**Neural substrates of smoking and reward cue reactivity in smokers: A meta-analysis of fMRI studies**

**Xiao Lin,^1, 2, #^ Jiahui Deng,^2, #^** **Le Shi,****^2^ Qiandong Wang,^1^ Peng Li,^2^ Hui Li,^2^ Jiajia Liu,^3^ Jianyu Que,****^2^ Suhua Chang,^2^ Yanping Bao,^3^ Jie Shi,****^3^ Daniel R. Weinberger,^4^**

**Ping Wu,^3,4,^ * Lin Lu^1, 2, 3,^** **^*^**

^1^Peking-Tsinghua Center for Life Sciences and PKU-IDG/McGovern Institute for Brain Research, Peking University, Beijing 100871, China

^2^Peking University Sixth Hospital, Peking University Institute of Mental Health, Key Laboratory of Mental Health, Ministry of Health (Peking University), National Clinical Research Center for Mental Disorders (Peking University Sixth Hospital), Beijing 100191, China

^3^National Institute on Drug Dependence and Beijing Key Laboratory of Drug Dependence, Peking University, Beijing 100191, China

^4^Lieber Institute for Brain Development and Departments of Psychiatry, Neurology, and Neuroscience and McKusick Nathans Institute of Genetic Medicine, Johns Hopkins University School of Medicine, Baltimore, Maryland 21205, USA

**^#^**These authors contributed equally

*These authors share senior authorship

*Corresponding author:

Prof. Lin Lu

Peking University Sixth Hospital, Peking University Institute of Mental Health, Key

Laboratory of Mental Health, Ministry of Health (Peking University), National Clinical

Research Center for Mental Disorders (Peking University Sixth Hospital), 51 Huayuan Bei Road, Haidian District, Beijing 100191, China

Tel: +86-10-82805308

Fax: +86-10-62032624

E-mail: [linlu@bjmu.edu.cn](mailto:linlu@bjmu.edu.cn)

Ping Wu, Ph.D.

National Institute on Drug Dependence and Beijing Key Laboratory of Drug Dependence, Peking University, 38 Xueyuan Road, Beijing 100191, China

Tel: +86-10-82802470-509

Fax: +86-10-62032624

E-mail: [wuping@bjmu.edu.cn](mailto:wuping@bjmu.edu.cn)

Running title: Neural mechanisms underlying reward processing in smokers

| **Supplementary Table S1. Details of the included studies, including demographic information.**  Part 1. For comparisons between smoking-related reward cues and neutral cues in smokers, 33 studies were identified (two studies reported brain reactivity to smoking-related cues in smokers and compared smokers and healthy controls; 26 studies only reported brain reactivity to smoking-related cues in smokers; five studies only reported comparisons between smokers and healthy controls). | | | | | | | | | | | | | |
| --- | --- | --- | --- | --- | --- | --- | --- | --- | --- | --- | --- | --- | --- |
| **Reference** | **Contrast**  **(abstinence)** | **Participants** | **Abstinence status** | **Sample size (female)** | **Ethnicity** | **Age (SD)** | **Years of education** | **Years of smoking** | **Cigarettes per day** | **Pack years (SD)** | **Age of smoking onset** | **FTND (SD)** | **Scanner** |
| 1 | a, b (1) | Smokers | > 2 h | 13 (5) | — | 26 (4) | — | — | 20.5 (3.4) | — | — | 5.4 (1.4) | 1.5 Tesla |
|  |  | Healthy controls | — | 13 (5) | — | 24 (4) | — | — |  | — | — | — |  |
| 2 | a (2) | Smokers | non-abstinent | 35 (0) | Chinese | 21.03 (1.22) | 13.89 (0.63) | 4.26 (2.27) | 14.83 (4.46) | 3.27 (2.56) | 15.29 (2.75) | 4.91 (1.69) | 3 Tesla |
|  |  | Healthy controls | — | 26 (0) | Chinese | 20.38 (1.32) | 13.58 (0.64) | — | — | — | — | — |  |
| 3 | a (2) | Smokers | 25 min abstinent | 42 (12) | — | 38 (12.4) | — | — | 23.3 (8.2) | 24.1 (24.5) | — | 5.7 (1.7) | 1.5 Tesla |
| 4 | a (1) | Smokers | 12 h abstinent | 17 (4) | 16 White  1 AA | 29.6 (9.4) | — | 12.2 (6.5) | 18 (7.10) | — | — | 4.94 (1.8) | 3 Tesla |
| 5 | a (2) | Smokers | non-abstinent | 15 (7) | — | — | — | — | 12 | — | — | > 5 | 3 Tesla |
| 6 | a (1) | Smokers | abstinent overnight | 9 (5) | 7 EA | 34.4 | — | — | 18.3 (8.7) | — | — | 4.7 (1.7) | 3 Tesla |
|  |  | Healthy controls |  | 11 (8) | 11 EA | 28.3 | — | — | — | — | — | — |  |
| 7 | a (2) | Smokers | non-abstinent | 30 (15) | 83% CC | 31.9 (9.4) | 12.9 (2.7) | 15.9 (9.6) | 19.1 (5.7) | — | 16.1 (3.4) | 4.2 (2.6) | 3 Tesla |
| 8 | a (2) | Smokers | non-abstinent | 22 (6) | 7 AA  14 EA  1 Mixed | 36.1 (2.2) | 14.2 (0.4) | — | 17.5 (1.6) | 12.6 (2.1) | — | 4.7 (0.4) | 3 Tesla |
| 9 | a (2) | Smokers | non-abstinent | 21 (10) | 12 CC  5 Hispanic / Latino  4 AA | 22.6 (2) | — | — | 11.1 (4.8) | — | — | 3.2 (1.75) | 3 Tesla |
| 10 | b | Smokers | — | 22 (6) | — | 38.95 (11.73) | 14.41 (1.76) | 15.5 (2.72) | 18.91 (10.3) | 16.41 (2.559) | — | 5.27 (2.53) | 3 Tesla |
|  |  | Healthy controls | — | 19 (10) | — | 39.63 (12.41) | 15.68 (2.26) | — | — | — | — | — |  |
| 11 | b (1) | Smokers | Overnight abstinent | 18 (0) | — | 33.8 (9.1) | 4.1 (1.1) | — | 17.2 (3.8) | — | — | 4 (1.5) | 3 Tesla |
|  |  | Healthy controls | — | 17 (0) | — | 34.7 (9.7) | 4.3 (1.2) | — | — | — | — | — |  |
| 12 | a (2) | Smokers | non-abstinent | 32 (19) | — | 33.5 (11.5) | — | — | 17.7 (6.9) | — | — | 5.6 (1.9) | 3 Tesla |
| 13 | a (1) | Smokers | 36 h abstinent | 23 (0) | — | 25 (1.2) | — | 6 (1.1) | 17.6 (0.8) | — | — | 5.5 (0.3) | 3 Tesla |
| 14 | a (1) | Smokers | 7 h abstinent | 8 (0) | — | 17.13 (0.76) | — | — | 15.33 (4.98) | — | — | 4.38 (2.13) | 1.5 Tesla |
| 15 | a (2) | Smokers | non-abstinent | 20 (10) | — | 27 (8) | — | — | 22 (6) | — | — | > 5 | 1.5 Tesla |
| 15 | a-repeated (1) | Same people | 12 h abstinent | — | — | — | — | — | — | — | — | — |  |
| 16 | a (1) | Smokers | 4 h abstinent | 29 (20) | — | 41.03 (9.05) | — | 22.02 (7.5) | 18.8 (4.32) | — | — | 6.62 (1.58) |  |
| 17 | a (1) | Smokers | 2 h abstinent | 58 (23) | 46 CC | 34.5 (1.6) | — | — | 16.8 (0.93) | — | — | 4.7 (0.27) | 3 Tesla |
| 18 | a (2) | Smokers | non-abstinent | 39 (15) | 25 CC  7 AA  3 Asian  4 Latino | 31.41 (8.81) | 14.61 (3.76) | — | 14.55 (7.45) | — | — | 5.15 (1.66) |  |
| 19 | b (1) | Healthy controls | — | 17 (9) | — | 24.59 (0.79) | — | — | — | — | — | — | 1.5 Tesla |
|  |  | Smokers | non-abstinent | 19 (10) |  | 27.21 (1.37) | — | 10.77 (1.17) | 20.79 (0.83) | — | — | 4.58 (0.49) | 1.5 Tesla |
|  |  | Smokers | Overnight smoking abstinent | 20 (10) |  | 25.8 (1.68) | — | 10.29 (1.75) | 19.43 (1.33) | — | — | 3.95 (0.39) |  |
| 20 | a, b (2) | Smokers | non-abstinent | 16 (8) | — | 25.75 (1.09) | — | 10.15 (1.3) | 19.81 (1.1) | — | — | 5.2 (0.58) | 1.5 Tesla |
|  |  | Healthy controls | — | 16 (8) | — | 23.31 (0.77) | — | — | — | — | — | — | 1.5 Tesla |
| 21 | a (2) | Smokers | non-abstinent | 35 (14) | All EA | 42.7 (11.3) | — | 23.4 (11.8) | 21.8 (10.6) | — | — | 4.6 (2.3) | 3 Tesla |
| 22 | a (2) | Smokers | non-abstinent | 22 (0) | — | 31 (7) | — | 14.6 (7.3) | 24.6 (7.2) | — | 16.8 (2.1) | 6.3 (1.5) | 1.5 Tesla |
| 23 | a (1) | Smokers | 12 h abstinent | 54 (17) | 25 AA  27 CC  2 Other | 45 (11.35) | — | — | > 10 | — | — | 5.03 (2.25) | 3 Tesla |
| 24 | a (1) | Smokers | 2 h abstinent | 90 (49) | 62 CC  23 AA  2 HA  3 Other | 29.9 (7.5) | 13.0 (2.2) | — | 20.3 (5.5) | — | — | 5 (1.6) | 3 Tesla |
| 25 | a (2) | Smokers | non-abstinent | 82 (12) | — | 33 (8.3) | 12.7 (1.7) | 15.6 (8.5) | 20.5 (5.6) | — | — | 4.9 (1.6) | 3 Tesla |
| 26 | a (2) | Smokers | non-abstinent | 10 (4) | — | — | — | 8.1 (5.5) | 13.7 (3.4) | — | — | 3.4 (1.6) | 3 Tesla |
| 27 | a (1) | Smokers | 24 h abstinent | 13 (13) | — | 43.2 (11.5) | — | > 6 months | > 10 | — | — | 6.3 (1.5) | 3 Tesla |
| 28 | a (2) | Smokers | non-abstinent | 17 (9) | — | 31.4 (6.1) | — | — | > 10 | 9.4 (5.5) | — | 5.6 (1.5) | 3 Tesla |
| 29 | a (1) | Smokers | 24 h abstinent | 30 (17) | 13 White  12 Black  5 Asian | 35.4 (11.5) | — | — | 14.9 (6.7) | — | — | 4.1 (1.9) | 3 Tesla |
| 30 | a (1) | Smokers | 2 h | 16 (13) | 4 Minority | 39.13 (9.89) | — | 20.9 (9.29) | 39.13  (9.89) | — | — | 6.5 (1.73) | 1.5 Tesla |
| 31 | a (2) | Smokers | — | 18 (10) | — | 25.06 (4.75) | 15.22 (2.08) | — | — | 6.38 (4.69) |  | 6.27 (1.02) | 3 Tesla |
| 32 | b (1) | Smokers | > 12 h abstinent | 12 (0) | — | 25.92 (1.83) | 19.17 (2.12) | 8.08 (1.78) | 21.3 (4.64) | — | — | 6.25 (0.97) | 3 Tesla |
|  |  | Healthy controls |  | 12 (0) | — | 26.08 (2.11) | 19.25 (2.01) | — | — | — | — | — |  |
| 33 | b (1) | Smokers | 4 h abstinent | 10 (4) | — | 31 (4.5) | — | 12.6 (4.7) | 14.8 (5.9) | 9.4 ( 6.5) | — | 3.8 (1.9) | 3 Tesla |
|  |  | Healthy controls |  | 10 (4) | — | 23 (1.8) | — | — | — |  | — | — |  |

Part 2. For non-smoking reward-related cue processing in smokers compared with healthy controls, 13 studies were identified.

| **Reference** | **Contrast** | **Participants** | **Sample size (female)** | **Ethnicity** | **Age (SD)** | **Years of education** | **Years of smoking** | **Cigarettes per day** | **Pack years** | **Age of smoking onset** | **FTND (SD)** | **Scanner** |
| --- | --- | --- | --- | --- | --- | --- | --- | --- | --- | --- | --- | --- |
| 34 | a | Smokers | 13 (6) | 12 CC  1 AA | 30.7 (9.7) | — | 13.5 (8.1) | 16.8 (4) | — | — | 4.7 (1.7) | 4.0 Tesla |
| 35 | b | Smokers | 21 | — | 28 (4.3) | — |  | 22 (3.5) | — | 16.2 (1.5) | 6.5 (1.2) | 3 Tesla |
|  |  | Healthy controls | 21 | — | 25.7 (6.1) | — | — | 0.5 (0.2) | — | 17.6 (2.8) | — |  |
| 36 | a | Smokers | 132 (52) | — | 31.36 | — | — | 14 (7.3) | — | — | 3.9 (2.7) | 3 Tesla |
| 37 | b | Smokers | 19 | — | 34.8 (9.8) | 4.2 (1.1) | — | > 15 | — | — | 5.1 (1.5) | 3 Tesla |
|  |  | Healthy controls | 19 | — | 34.1 (9.3) | — | — | — | — | — | — |  |
| 38 | b | Smokers | 23 (11) |  | 35 (11.2) | — | 16.5 (10.14) | 15.26 (5.96) | — | — | 57.12  (11.3)  NDSS****** | 3 Tesla |
|  |  | Healthy controls | 11 (6) |  | 35.3 (14.79) | — | — | — | — | — | — |  |
| 39 | a | Smokers | 42 (0) | 32 CC  9 AA  1 Asian | 26.6 (7.1) | — | — | 15.9 (7.5) | — | — | 3.1 (1.9) | 3 Tesla |
| 40 | b | Smokers | 15 |  | 25.6 (2.1) | — | — | 17.2 (1.5) | — | — | 4.3 (0.3) | 3 Tesla |
|  |  | Healthy controls | 17 |  | 21.2 (0.8) | — | — | 0.06 (0.04) | — | — | — |  |
| 41 | b | Smokers | 34 (18) | 1 CC  2 AA  19 HA  1 American Indian / Alaskan native  11 Multiracial | 16.29 (1.2) | — | — | 5.99 (7.2) | — | — | — | 3 Tesla |
|  |  | Healthy controls | 38 (14) | 4 CC  26 HA  7 Multiracial | 15.76 (1.2) | — | — | — |  |  | — |  |
| 42 | b | Smokers | 31 (13) | — | 41.3 (7.9) | — | — | 24.5 (6.6) |  |  | 6.1 (1.7) | 3 Tesla |
|  |  | Healthy controls | 35 (19) | — | 41.3 (7.9) | — | — | — |  |  | — |  |
| 43 | b | Smokers | 19 (13) | — | 26.89 (8.11) | — | 9.19 (4.16) | 18.95 (4.4) |  |  | 3.79 (2.1) | 3 Tesla |
|  |  | Healthy controls | 17 (10) | — | 24.47 (7.79) | — | — | — |  |  | — |  |
| 44 | a | Smokers | 13 (6) | 9 White  1 Black  1 Native American / Pacific Islander  2 Asian | 40.15 (13.1) | — | — | 22.31 (8.55) |  |  | 6.54 (1.2) | 3 Tesla |
| 45 | b | Smokers | 16 (10) | — | 31.4 (9.82) | — | — | 15.17 (4.91) |  |  | 3.56 (1.9) | 4 Tesla |
|  |  | Healthy controls | 17 (9) | — | 33.73 (10.29) | — | — | — |  |  | — |  |
| 46 | b | Smokers | 15 (7) | — | 23.3 (1.2) | 16.5 (0.5) | 7.1 (1.3) | 16 (1.2) | 6.2 (1.5) | — | 3.5 (0.2) | 3 Tesla |
|  |  | Healthy controls | 15 (9) | — | 23.8 (1.2) | 18.15 (0.4) | — | — | — | — | — |  |
| 47 | b | Smokers | 19 (14) | — | 30.42 (3.45) | 13.74 (1.73) | — | 10.02 (4.57) | — | — | — | 3 Tesla |
|  |  | Healthy controls | 19 (14) | — | 29.95 (3.39) | 14.53 (1.74) | — | — | — | — | — |  |
| 48 | b | Smokers | 21 (13) | 7 AA  14 CC | 32.9 (9.77) | 12.9 (2.7) | 16.36 | 22.55 (5.6) | 15.62 |  | 5.76 | 3 Tesla |
|  |  | Healthy controls | 21 (9) | 6 AA  14 CC  1 Asian | 30.29 (8.74) | 13.94 (2.54) | — | — |  |  | — |  |
| 49 | b | Smokers | 28 (15) | 8 AA  20 CC | 32.68 (10.02) | 12.89 (2.49) | 16.48 | 22.8 |  | 15.46 | 5.89 | 3 Tesla |
|  |  | Healthy controls | 28 (12) | 11 AA  14 CC  3 Asian | 30.11 (7.83) | 14 (2.61) | — | — | — | — | — |  |
| 50 | b | Smokers | 14 | — | 25 (4.5) | 14 (1.2) | — | 13.2 (6.4) | — | — | — | 1.5 Tesla |
|  |  | Healthy controls | 13 | — | 24 (2.7) | 15 (1.2) | — | — | — | — | — |  |
| 51 | a | Smokers | 44 (20) | — | 26.1 (7) | — | — | 15.4 (3.4) | — | — | 4 (1.7) | 3 Tesla |

CC, Caucasian; AA, African American; EA, European American; HA, Hispanic American; NDSS, Nicotine Dependence Syndrome Scale; FTND, Fagerstrom Test for Nicotine Dependence. ^a^ One-sample contrast. ^b^ Two-sample contrast. ^(1)^ Abstinent. ^(2)^ Non-abstinent. ** FTND scores not provided (i.e., in the meta-regression, the severity of nicotine dependence was null).

**Supplementary Table S2. Suprathreshold clusters from activation likelihood estimation meta-analysis (data from studies included abstinent smokers in 12 studies**^(1)^ **in Supplementary Table S1).**

| **Brain region** | **MNI coordinates** | **SDM-Z** | ***p*** | **Voxels** |
| --- | --- | --- | --- | --- |
| Left anterior cingulate / paracingulate gyri, BA 32 | -4, 46, 10 | 3.803 | 0.000010550 | 2581 |
| Right median cingulate / paracingulate gyri, BA 23 | 4, -36, 34 | 3.758 | 0.000012279 | 2380 |
| Right lenticular nucleus, putamen | 30, 6, 2 | 2.696 | 0.001177609 | 261 |
| Right inferior network, inferior longitudinal fasciculus | 28, -66, -6 | -1.083 | 0.001456380 | 145 |
| Right inferior network, inferior longitudinal fasciculus | -58, -16, -4 | -1.038 | 0.001730919 | 103 |

**Supplementary Table S3. Suprathreshold clusters from activation likelihood estimation meta-analysis (data from studies included satiated smokers in 16 studies^(2)^ in Supplementary Table S1).**

| **Brain region** | **MNI coordinates** | **SDM-Z** | ***p*** | **Voxels** |
| --- | --- | --- | --- | --- |
| Right anterior cingulate / paracingulate gyri, BA 32 | 4, 44, 16 | 4.540 | 0.000045180 | 2891 |
| Right precuneus, BA 23 | 4, -60, 26 | 5.138 | 0.000661910 | 2393 |
| Right striatum | 6, 10, 0 | 3.061 | 0.002239883 | 157 |
| Left anterior thalamic projections | -10, -8, 8 | 2.819 | 0.002992988 | 31 |
| Left median cingulate / paracingulate gyri | -2, -6, 46 | 2.754 | 0.000106514 | 19 |
| Right inferior frontal gyrus, opercular part | 44, 8, 26 | -1.132 | 0.000385046 | 432 |
| Left postcentral gyrus, BA 4 | -54, -20, 50 | -1.107 | 0.000385046 | 98 |

**Supplementary Table S4. Meta-regression results of brain regions that showed different reactivation in response to smoking-related cues between smokers with maximum FDNT scores and smokers with minimum FDNT scores.**

| **Brain region** | **MNI coordinates** | **SDM-Z** | ***p*** | **Voxels** |
| --- | --- | --- | --- | --- |
| Right inferior occipital gyrus, BA 19 | 40, -80, -6 | 2.917 | 0.000033319 | 397 |
| Right calcarine fissure / surrounding cortex, BA 17 | 4, -70, 10 | 1.924 | 0.003898084 | 17 |
| Right striatum | 12, 16, -2 | -2.039 | 0.000444114 | 174 |
| Left middle temporal gyrus, BA 37 | -50, -66, -4 | -1.913 | 0.000795603 | 105 |
| Right postcentral gyrus, BA 2 | 40, -44, 62 | -1.850 | 0.001076758 | 91 |
| Right supramarginal gyrus, BA 48 | 48, -28, 24 | -1.673 | 0.002329051 | 45 |
| Left postcentral gyrus, BA 48 | -62, -20, 22 | -1.603 | 0.003137589 | 36 |
| Right supramarginal gyrus, BA 2 | 64, -28, 40 | -1.699 | 0.002084374 | 27 |

**Supplementary Table S5. Meta-regression results of brain regions that showed different reactivation in response to non-smoking reward-related cues between smokers with maximum FDNT scores and smokers with minimum FDNT scores.**

| **Brain region** | **MNI coordinates** | **SDM-Z** | ***p*** | **Voxels** |
| --- | --- | --- | --- | --- |
| Right anterior cingulate / paracingulate gyri, BA 24 | 4, 14, 26 | 1.370 | 0.000205517 | 536 |
| Left inferior network, inferior longitudinal fasciculus | -28, -10, -14 | 1.375 | 0.000186801 | 241 |
| Left striatum | -8, 12, -10 | -1.755 | 0.000000298 | 700 |
| Right inferior parietal (excluding supramarginal and angular) gyri, BA 40 | 42, -52, 46 | -1.066 | 0.001068592 | 287 |
| Right frontal inferior longitudinal fasciculus | 46, 20, 22 | -1.086 | 0.000261128 | 156 |
| Right insula, BA 47 | 34, 18, -8 | -1.022 | 0.001628757 | 35 |

**Supplementary Table S6. Results of Jackknife sensitivity analyses of brain reactivity to smoking-related cues in smokers.**

| **Excluded study in Jackknife sensitivity analyses** | **Left anterior cingulate / paracingulate gyri, BA 32** |  | **Left angular gyrus, BA 39** | **Right thalamus** | **Corpus callosum** | **Right striatum** | **Left middle temporal gyrus, BA 22** |
| --- | --- | --- | --- | --- | --- | --- | --- |
| Artiges et al., 2009 | 1 | 1 | | 1 | 1 | 1 | 1 |
| Bi et al., 2017 | 1 | 1 | | 1 | 1 | 1 | 1 |
| Brody et al., 2007 | 1 | 0 | | 1 | 1 | 1 | 1 |
| Cortesea et al., 2015 | 1 | 1 | | 1 | 1 | 1 | 1 |
| Dagher et al., 2009 | 1 | 1 | | 1 | 1 | 1 | 1 |
| David et al., 2005 | 1 | 1 | | 1 | 1 | 1 | 1 |
| Dinh-Williams et al., 2014 | 1 | 1 | | 1 | 1 | 1 | 1 |
| Franklin et al., 2011 | 1 | 1 | | 1 | 1 | 1 | 0 |
| Ghahremani et al., 2018 | 1 | 1 | | 1 | 1 | 1 | 1 |
| Hartwell et al., 2011 | 1 | 1 | | 1 | 1 | 1 | 1 |
| Janes et al., 2009 | 1 | 1 | | 1 | 1 | 1 | 1 |
| Janes et al., 2015a | 1 | 1 | | 1 | 1 | 1 | 1 |
| Janes et al., 2015b | 1 | 1 | | 1 | 1 | 1 | 1 |
| Kang et al., 2012 | 1 | 1 | | 1 | 1 | 1 | 1 |
| Lee et al., 2005 | 1 | 1 | | 1 | 1 | 1 | 1 |
| McBride et al., 2006 | 1 | 1 | | 1 | 1 | 1 | 0 |
| McClernon et al., 2007 | 1 | 1 | | 1 | 1 | 1 | 1 |
| McClernon et al., 2016 | 1 | 1 | | 1 | 1 | 1 | 1 |
| Mondino et al., 2018 | 1 | 1 | | 1 | 1 | 1 | 1 |
| Moran-Santa et al., 2015 | 1 | 1 | | 1 | 1 | 1 | 1 |
| Ray et al., 2015 | 1 | 1 | | 1 | 1 | 1 | 1 |
| Stippekohl et al., 2012 | 1 | 1 | | 1 | 1 | 1 | 1 |
| Versace et al., 2011 | 1 | 1 | | 1 | 0 | 1 | 1 |
| Vollstadt et al., 2011 | 1 | 1 | | 1 | 1 | 1 | 1 |
| Westbrook et al., 2013 | 1 | 1 | | 1 | 1 | 1 | 1 |
| Wilson et al., 2012 | 1 | 0 | | 1 | 0 | 0 | 1 |
| Wilson et al., 2013 | 1 | 1 | | 0 | 0 | 0 | 0 |
| Total | 28/28 | 26/28 | | 27/28 | 25/28 | 26/28 | 25/28 |

**Supplementary Table S7. Results of Jackknife sensitivity analyses of brain reactivity to smoking-related cues in smokers compared with healthy controls.**

| **Excluded study in Jackknife sensitivity analyses** | **Right middle frontal gyrus, BA 46** |  | **Right middle frontal gyrus, orbital part, BA 11** | **Right lenticular nucleus, putamen** | **Left superior frontal gyrus, dorsolateral, BA 6** |
| --- | --- | --- | --- | --- | --- |
| Artiges et al., 2009 | 1 | | 1 | 1 | 1 |
| Gilman et al., 2018 | 1 | | 1 | 1 | 1 |
| Goudriann et al., 2010 | 1 | | 1 | 1 | 1 |
| Lee et al., 2012 | 1 | | 1 | 1 | 1 |
| Stippekohl et al., 2012 | 0 | | 0 | 0 | 1 |
| Yalachkov et al., 2013 | 1 | | 1 | 1 | 1 |
| Stippekohl et al., 2010 | 1 | | 1 | 1 | 1 |
| Total | 6/7 | | 6/7 | 6/7 | 7/7 |

**Supplementary Table S8. Results of Jackknife sensitivity analyses of brain reactivity to nonsmoking reward-related cues in smokers compared with healthy controls.**

| **Excluded studies in Jackknife sensitivity analyses** | **Right inferior parietal (excluding supramarginal and angular) gyri, BA 40** | **Right insula, BA 47** | **Right inferior frontal gyrus, opercular part, BA 44** | **Left striatum** |
| --- | --- | --- | --- | --- |
| Peechatka et al., 2017 | 1 | 1 | 1 | 1 |
| Buhler et al., 2010 | 1 | 1 | 1 | 1 |
| Geier et al., 2014 | 1 | 1 | 1 | 1 |
| Van Hell et al., 2010 | 1 | 1 | 1 | 1 |
| Jansma et al., 2013 | 1 | 1 | 1 | 1 |
| Karoly et al., 2015 | 1 | 1 | 1 | 1 |
| Kobiella et al., 2014 | 1 | 1 | 1 | 1 |
| Nector et al., 2018 | 1 | 1 | 1 | 1 |
| Luijten et al., 2013 | 0 | 0 | 0 | 1 |
| Martin et al., 2014 | 1 | 1 | 1 | 1 |
| De Ruiter et al., 2009 | 0 | 0 | 0 | 1 |
| Rose et al., 2012 | 1 | 1 | 1 | 1 |
| Rose et al., 2013 | 1 | 1 | 1 | 1 |
| Total | 12/13 | 12/13 | 12/13 | 13/13 |

**Figure S1. Funnel plots of publication bias of fMRI studies for processing smoking-related cues and nonsmoking reward-related cues.**


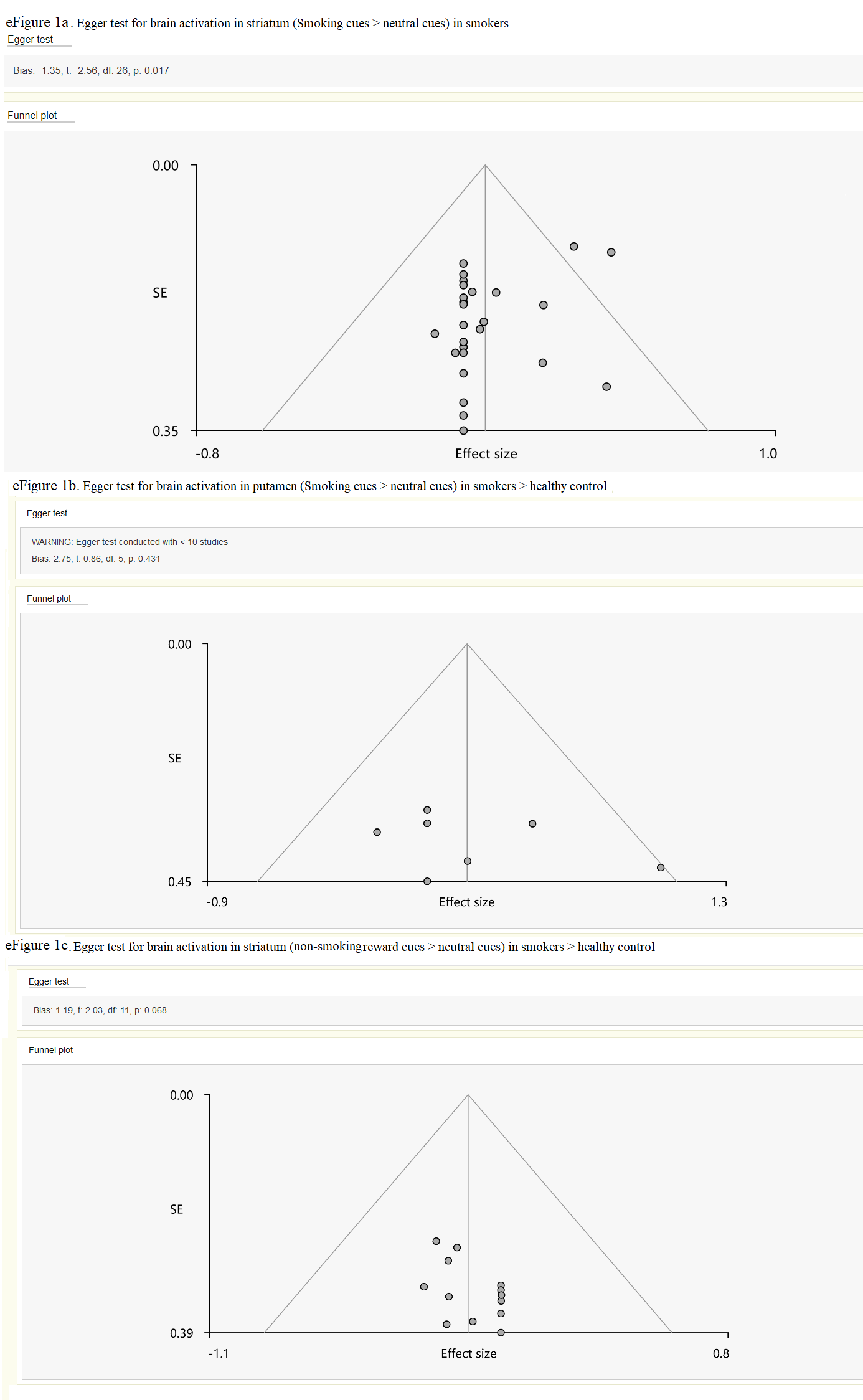


**References**

1. Artiges E, et al. Exposure to smoking cues during an emotion recognition task can modulate limbic fMRI activation in cigarette smokers. *Addict. Biol.* **14**, 469-477 (2009).

2. Bi Y, et al. White matter integrity of central executive network correlates with enhanced brain reactivity to smoking cues. *Hum. Brain Mapp.* **38**, 6239-6249 (2017).

3. Brody AL, et al. Neural substrates of resisting craving during cigarette cue exposure. *Biol. Psychiatry* **62**, 642-651 (2007).

4. Cortese BM, et al. The fMRI BOLD response to unisensory and multisensory smoking cues in nicotine-dependent adults. *Psychiatry Res.* **234**, 321-327 (2015).

5. Dagher A, Tannenbaum B, Hayashi T, Pruessner JC, McBride D. An acute psychosocial stress enhances the neural response to smoking cues. *Brain Res.* **1293**, 40-48 (2009).

6. David SP, et al. Ventral striatum/nucleus accumbens activation to smoking-related pictorial cues in smokers and nonsmokers: a functional magnetic resonance imaging study. *Biol. Psychiatry* **58**, 488-494 (2005).

7. Dinh-Williams L, Mendrek A, Bourque J, Potvin S. Where there's smoke, there's fire: the brain reactivity of chronic smokers when exposed to the negative value of smoking. *Prog. Neuropsychopharmacol. Biol. Psychiatry* **50**, 66-73 (2014).

8. Franklin T, et al. Effects of varenicline on smoking cue-triggered neural and craving responses. *Arch. Gen. Psychiatry* **68**, 516-526 (2011).

9. Ghahremani DG, Faulkner P, Cox CM, London ED. Behavioral and neural markers of cigarette-craving regulation in young-adult smokers during abstinence and after smoking. *Neuropsychopharmacology* **43**, 1616-1622 (2018).

10. Gilman JM, et al. Anterior insula activation during inhibition to smoking cues is associated with ability to maintain tobacco abstinence. *Addict. Behav. Rep.* **7**, 40-46 (2018).

11. Goudriaan AE, De Ruiter MB, Van Den Brink W, Oosterlaan J, Veltman DJ. Brain activation patterns associated with cue reactivity and craving in abstinent problem gamblers, heavy smokers and healthy controls: an fMRI study. *Addict. Biol.* **15**, 491-503 (2010).

12. Hartwell KJ, et al. Neural correlates of craving and resisting craving for tobacco in nicotine dependent smokers. *Addict. Biol.* **16**, 654-666 (2011).

13. Kang OS, et al. Individual differences in smoking-related cue reactivity in smokers: an eye-tracking and fMRI study. *Prog. Neuropsychopharmacol. Biol. Psychiatry* **38**, 285-293 (2012).

14. Lee JH, Lim Y, Wiederhold BK, Grahama SJ. A functional magnetic resonance imaging (fMRI) study of cue-induced smoking craving in yirtual environments. *Appl. Psychophysiol. Biofeedback* **30**, 195-204 (2005).

15. McBride D, Barrett SP, Kelly JT, Aw A, Dagher A. Effects of expectancy and abstinence on the neural response to smoking cues in cigarette smokers: an fMRI study. *Neuropsychopharmacology* **31**, 2728-2738 (2006).

16. Mondino M, et al. Effects of repeated transcranial direct current stimulation on smoking, craving and brain reactivity to smoking cues. *Sci. Rep.* **8**, 8724 (2018).

17. Moran-Santa Maria MM, et al. Right anterior insula connectivity is important for cue-induced craving in nicotine-dependent smokers: insula connectivity in smokers. *Addict. Biol.* **20**, 407-414 (2015).

18. Ray LA, et al. Varenicline, naltrexone, and their combination for heavy-drinking smokers: preliminary neuroimaging findings. *Am. J. Drug Alcohol Abuse* **41**, 35-44 (2015).

19. Stippekohl B, et al. Neural responses to BEGIN- and END-stimuli of the smoking ritual in nonsmokers, nondeprived smokers, and deprived smokers. *Neuropsychopharmacology* **35**, 1209-1225 (2010).

20. Stippekohl B, et al. Neural responses to smoking stimuli are influenced by smokers' attitudes towards their own smoking behaviour. *PLoS One* **7**, e46782 (2012).

21. Versace F, et al. Do brain responses to emotional images and cigarette cues differ? An fMRI study in smokers. *Eur. J. Neurosci.* **34**, 2054-2063 (2011).

22. Vollstädt-Klein S, et al. Severity of dependence modulates smokers' neuronal cue reactivity and cigarette craving elicited by tobacco advertisement: neural response to tobacco advertisement. *Addict. Biol.* **16**, 166-175 (2011).

23. Westbrook C, et al. Mindful attention reduces neural and self-reported cue-induced craving in smokers. *Soc. Cogn. Affect. Neurosci.* **8**, 73-84 (2013).

24. Wilson SJ, Sayette MA, Fiez JA. Quitting-unmotivated and quitting-motivated cigarette smokers exhibit different patterns of cue-elicited brain activation when anticipating an opportunity to smoke. *J. Abnorm. Psychol.* **121**, 198-211 (2012).

25. Wilson SJ, Creswell KG, Sayette MA, Fiez JA. Ambivalence about smoking and cue-elicited neural activity in quitting-motivated smokers faced with an opportunity to smoke. *Addict. Behav.* **38**, 1541-1549 (2013).

26. Janse Van Rensburg K, Taylor A, Hodgson T, Benattayallah A. Acute exercise modulates cigarette cravings and brain activation in response to smoking-related images: an fMRI study. *Psychopharmacology* **203**, 589-598 (2009).

27. Janes AC, et al. Brain fMRI reactivity to smoking-related images before and during extended smoking abstinence. *Exp. Clin. Psychopharmacol.* **17**, 365-373 (2009).

28. Janes AC, Farmer S, Peechatka AL, Frederick Bde B, Lukas SE. Insula-dorsal anterior cingulate cortex coupling is associated with enhanced brain reactivity to smoking cues. *Neuropsychopharmacology* **40**, 1561-1568 (2015).

29. McClernon FJ, et al. Hippocampal and insular response to smoking-related environments: neuroimaging evidence for drug-context effects in nicotine dependence. *Neuropsychopharmacology* **41**, 877-885 (2016).

30. McClernon FJ, Hutchison KE, Rose JE, Kozink RV. DRD4 VNTR polymorphism is associated with transient fMRI-BOLD responses to smoking cues. *Psychopharmacology* **194**, 433-441 (2007).

31. Janes AC, et al. Memory retrieval of smoking-related images induce greater insula activation as revealed by an fMRI-based delayed matching to sample task. *Addict. Biol.* **20**, 349-356 (2015).

32. Lee JH, Kim DY, Kim J. Mesocorticolimbic hyperactivity of deprived smokers and brain imaging. *Neuroreport* **23**, 1039-1043 (2012).

33. Yalachkov Y, Kaiser J, Görres A, Seehaus A, Naumer MJ. Sensory modality of smoking cues modulates neural cue reactivity. *Psychopharmacology* **225**, 461-471 (2013).

34. Addicott MA, et al. Smoking withdrawal is associated with increases in brain activation during decision making and reward anticipation: a preliminary study. *Psychopharmacology* **219**, 563-573 (2012).

35. Buhler M, et al. Nicotine dependence is characterized by disordered reward processing in a network driving motivation. *Biol. Psychiatry* **67**, 745-752 (2010).

36. Claus ED, Blaine SK, Filbey FM, Mayer AR, Hutchison KE. Association between nicotine dependence severity, BOLD response to smoking cues, and functional connectivity. *Neuropsychopharmacology* **38**, 2363-2372 (2013).

37. de Ruiter MB, et al. Response perseveration and ventral prefrontal sensitivity to reward and punishment in male problem gamblers and smokers. *Neuropsychopharmacology* **34**, 1027-1038 (2009).

38. Geier CF, Sweitzer MM, Denlinger R, Sparacino G, Donny EC. Abstinent adult daily smokers show reduced anticipatory but elevated saccade-related brain responses during a rewarded antisaccade task. *Psychiatry Res.* **223**, 140-147 (2014).

39. Gray JC, et al. Clarifying the neural basis for incentive salience of tobacco cues in smokers. *Psychiatry Res.* **223**, 218-225 (2014).

40. Jansma JM, et al. THC reduces the anticipatory nucleus accumbens response to reward in subjects with a nicotine addiction. *Transl. Psychiatry* **3**, e234 (2013).

41. Karoly HC, et al. Does incentive-elicited nucleus accumbens activation differ by substance of abuse? An examination with adolescents. *Dev. Cogn. Neurosci.* **16**, 5-15 (2015).

42. Kobiella A, et al. Acute and chronic nicotine effects on behaviour and brain activation during intertemporal decision making: discounting under nicotine. *Addict. Biol.* **19**, 918-930 (2014).

43. Luijten M, O'Connor DA, Rossiter S, Franken IH, Hester R. Effects of reward and punishment on brain activations associated with inhibitory control in cigarette smokers. *Addiction* **108**, 1969-1978 (2013).

44. MacKillop J, et al. The neuroeconomics of nicotine dependence: a preliminary study of delay discounting of monetary and cigarette rewards in smokers using fMRI. *Psychiatry Res.* **202**, 20-29 (2012).

45. Martin LE, Cox LS, Brooks WM, Savage CR. Winning and losing: differences in reward and punishment sensitivity between smokers and nonsmokers. *Brain Behav.* **4**, 915-924 (2014).

46. Nestor LJ, McCabe E, Jones J, Clancy L, Garavan H. Shared and divergent neural reactivity to non-drug operant response outcomes in current smokers and ex-smokers. *Brain Res.* **1680**, 54-61 (2018).

47. Peechatka AL, Janes AC. Association between reward reactivity and drug use severity is substance dependent: preliminary evidence from the Human Connectome Project. *Nicotine Tob. Res.* **19**, 710-715 (2017).

48. Rose EJ, et al. Chronic exposure to nicotine is associated with reduced reward-related activity in the striatum but not the midbrain. *Biol. Psychiatry* **71**, 206-213 (2012).

49. Rose EJ, et al. Acute nicotine differentially impacts anticipatory valence- and magnitude-related striatal activity. *Biol. Psychiatry* **73**, 280-288 (2013).

50. van Hell HH, et al. Chronic effects of cannabis use on the human reward system: an fMRI study. *Eur. Neuropsychopharmacology* **20**, 153-163 (2010).

51. Wilson SJ, et al. Weak ventral striatal responses to monetary outcomes predict an unwillingness to resist cigarette smoking. *Cogn. Affect. Behav. Neurosci.* **14**, 1196-1207 (2014).
